# Prevalence of Gastrointestinal Symptoms in Severe Acute Respiratory Syndrome Coronavirus 2 Infection: Results of the Prospective Controlled Multinational GI-COVID-19 Study

**DOI:** 10.14309/ajg.0000000000001541

**Published:** 2021-11-09

**Authors:** Giovanni Marasco, Cesare Cremon, Maria Raffaella Barbaro, Daniele Salvi, Giulia Cacciari, Anna Kagramanova, Dmitry Bordin, Vasile Drug, Edgidia Miftode, Pietro Fusaroli, Salem Youssef Mohamed, Chiara Ricci, Massimo Bellini, M. Masudur Rahman, Luigi Melcarne, Javier Santos, Beatriz Lobo, Serhat Bor, Suna Yapali, Deniz Akyol, Ferdane Pirincci Sapmaz, Yonca Yilmaz Urun, Tugce Eskazan, Altay Celebi, Huseyin Kacmaz, Berat Ebik, Hatice Cilem Binicier, Mehmet Sait Bugdayci, Munkhtsetseg Banzragch Yağcı, Husnu Pullukcu, Berrin Yalınbas Kaya, Ali Tureyen, İbrahim Hatemi, Elif Sitre Koc, Goktug Sirin, Ali Riza Calıskan, Goksel Bengi, Esra Ergun Alıs, Snezana Lukic, Meri Trajkovska, Keren Hod, Dan Dumitrascu, Antonello Pietrangelo, Elena Corradini, Magnus Simren, Jessica Sjolund, Navkiran Tornkvist, Uday C. Ghoshal, Olga Kolokolnikova, Antonio Colecchia, Jordi Serra, Giovanni Maconi, Roberto De Giorgio, Silvio Danese, Pietro Portincasa, Michele Di Stefano, Marcello Maggio, Elena Philippou, Yeong Yeh Lee, Alessandro Venturi, Claudio Borghi, Marco Zoli, Paolo Gionchetti, Pierluigi Viale, Vincenzo Stanghellini, Giovanni Barbara

**Affiliations:** 1 IRCCS Azienda Ospedaliero-Universitaria di Bologna, Bologna, Italy;; 2 Department of Medical and Surgical Sciences, University of Bologna, Italy;; 3 Moscow Clinical Scientific Center, Moscow, Russia;; 4 A. S. Loginov Moscow Clinical Scientific Center, Moscow, Russia;; 5 Tver State Medical University, Tver, Russia;; 6 A.I. Yevdokimov Moscow State University of Medicine and Dentistry, Moscow, Russia;; 7 Department of Gastroenterology, “Grigore T. Popa” University of Medicine and Pharmacy, Iasi, Romania;; 8 Department of Infectious Diseases, “Grigore T. Popa” University of Medicine and Pharmacy, Iasi, Romania;; 9 Gastroenterology Unit, Imola Hospital, Imola, Italy;; 10 Gastroenterology and Hepatology Unit, Internal Medicine Department, Faculty of Medicine, Zagazig University, Egypt;; 11 Department of Experimental and Clinical Sciences, University of Brescia, Spedali Civili di Brescia, Brescia, Italy;; 12 Gastroenterology Unit, University of Pisa, Pisa, Italy;; 13 Sheikh Russel National Gastroliver Institute and Hospital, Dhaka, Bangladesh;; 14 Hospital Universitari Parc Taulí, Sabadell - CIBEREHD Centro de Investigación Biomédica en Red, Spain;; 15 Hospital General Vall d’Hebron, Barcelona, Spain;; 16 Division of Gastroenterology, Ege University, Izmir, Turkey;; 17 Division of Gastroenterology, Acibadem University, Altunizade Acibadem Hospital, Istanbul, Turkey;; 18 Ege University Department of Infectious Diseases, Izmir, Turkey;; 19 University of Health Sciences, Keciören Education and Research Hospital, Division of Gastroenterology, Keciören, Turkey;; 20 Division of Gastroenterology, Eskisehir City Hospital, Eskisehir, Turkey;; 21 Istanbul University-Cerrahpasa, Cerrahpasa Faculty of Medicine, Division of Gastroenterology, Turkey;; 22 Division of Gastroenterology, Kocaeli University, Kocaeli, Turkey;; 23 Division of Gastroenterology, Adiyaman Education and Research Hospital, Adiyaman, Turkey;; 24 Division of Gastroenterology, University of Health Sciences, Diyabakır Gazi Yasargil Education and Research Hospital, Diyarbakır, Turkey;; 25 Division of Gastroenterology, Dokuz Eylül University, Izmir, Turkey;; 26 Division of Gastroenterology, İstanbul Aydın University Florya Liv Hospital, Istanbul, Turkey;; 27 Division of Gastroenterology, Darıca Farabi Education and Research Hospital, Kocaeli, Turkey;; 28 Department of Infectious Diseases, İstanbul Aydın University Florya Liv Hospital, Istanbul, Turkey;; 29 University Clinical Centre of Serbia, Clinic for Gastroenterohepatology, Belgrade, Serbia;; 30 Clinic of Gastroenterohepatology, Skopje, North Macedonia;; 31 Assuta Medical Centers, Tel Aviv, Israel;; 32 Iuliu Hatieganu University of Medicine and Pharmacy, Cluj-Napoca Romania;; 33 Internal Medicine Unit, Modena University Hospital, University of Modena and Reggio Emilia, Modena, Italy;; 34 Sahlgrenska University Hospital, Gothenburg, Sweden;; 35 Sanjay Gandhi Postgraduate Institute of Medical Sciences, Lucknow, India;; 36 Medsi Clinical Hospital, Russia;; 37 Gastroenterology Unit, Verona University Hospital, Verona, Italy;; 38 CIBERehd, University Hospital Germans Trias i Pujol, Barcelona, Spain;; 39 Sacco Hospital, University of Milan, Milan, Italy;; 40 University of Ferrara, Ferrara, Italy;; 41 Humanitas Hospital, Milan, Italy;; 42 Internal Medicine Unit, Bari University Hospital, Bari, Italy;; 43 First Department of Internal Medicine, Fondazione IRCCS Policlinico San Matteo, University of Pavia, Pavia, Italy;; 44 Geriatric Clinic Unit, Medical Geriatric Rehabilitative Department, University Hospital of Parma, Parma, Italy;; 45 Department of Life and Health Sciences, Cyprus University of Nicosia, Cyprus;; 46 School of Medical Sciences, Universiti Sains Malaysia, Kota Bharu, Malaysia.

## Abstract

Gastrointestinal (GI) symptoms in coronavirus-19 disease (COVID-19) have been reported with great variability and without standardization. In hospitalized patients, we aimed to evaluate the prevalence of GI symptoms, factors associated with their occurrence, and variation at 1 month.

**METHODS:**

The GI-COVID-19 is a prospective, multicenter, controlled study. Patients with and without COVID-19 diagnosis were recruited at hospital admission and asked for GI symptoms at admission and after 1 month, using the validated Gastrointestinal Symptom Rating Scale questionnaire.

**RESULTS:**

The study included 2036 hospitalized patients. A total of 871 patients (575 COVID+ and 296 COVID−) were included for the primary analysis. GI symptoms occurred more frequently in patients with COVID-19 (59.7%; 343/575 patients) than in the control group (43.2%; 128/296 patients) (*P* < 0.001). Patients with COVID-19 complained of higher presence or intensity of nausea, diarrhea, loose stools, and urgency as compared with controls. At a 1-month follow-up, a reduction in the presence or intensity of GI symptoms was found in COVID-19 patients with GI symptoms at hospital admission. Nausea remained increased over controls. Factors significantly associated with nausea persistence in COVID-19 were female sex, high body mass index, the presence of dyspnea, and increased C-reactive protein levels.

**DISCUSSION:**

The prevalence of GI symptoms in hospitalized patients with COVID-19 is higher than previously reported. Systemic and respiratory symptoms are often associated with GI complaints. Nausea may persist after the resolution of COVID-19 infection.

## INTRODUCTION

The clinical course of severe acute respiratory syndrome coronavirus 2 (SARS-CoV-2) infection, known as coronavirus-19 disease (COVID-19), can range from asymptomatic to rapidly progressing and life-threatening disease. Severe COVID-19 is characterized by hypoxemia, respiratory insufficiency, and eventually multiple organ failure (1). SARS-CoV-2 may also infect the gastrointestinal (GI) tract (2), for which it has a marked tropism. The virus binds to angiotensin-converting enzyme 2 receptors, which are abundantly expressed on epithelial cells, causing local and systemic inflammation (3,4). GI tract involvement is associated with nausea, vomiting, abdominal pain, and diarrhea (5). Stool shedding of SARS-CoV-2 RNA in approximately 50% of patients with COVID-19 confirms that the GI tract is a target organ of the virus and raises concerns about possible oral-fecal spread of the disease (6). A meta-analysis (7) of 29 studies, including 4,805 patients with COVID, reported a pooled prevalence of nausea/vomiting and diarrhea of 4.6% and 7.4%, respectively. However, the results were highly heterogeneous, with symptom prevalence ranging between 3.4% and 17.0% (8,9). In addition, most studies were mainly obtained from the same COVID-19 clusters; they were frequently uncontrolled, retrospective or observational, and data were collected without the aid of validated questionnaires. Thus, attempts to evaluate the prevalence of GI symptoms in COVID-19 and the risk of GI symptoms for respiratory failure or mortality based on these data have led to inconclusive results (10). We conducted a global, multicenter, controlled study to determine the prevalence of GI symptoms in hospitalized patients with COVID-19 compared with a non-COVID control group. In addition, we assessed risk factors for the development of COVID-19–related GI symptoms and the persistence of GI symptoms 1 month after the initial assessment.

## METHODS

### Oversight

This study was promoted by GB, GM, CC, and VS at the Department of Medical and Surgical Science, University of Bologna, Italy, and IRCCS S. Orsola in Bologna, Italy. The study was endorsed by the United European Gastroenterology, the European Society of Neurogastroenterology and Motility, and the Rome Foundation. All patients were clinically evaluated according to standard clinical practice. The study was conducted according to the Declaration of Helsinki for clinical studies and approved by each local ethics committee (approval identification number: 399/2020/Oss/AOUBo). Written informed consent was obtained from all subjects.

### Patients

Eligible patients were ≥18 and ≤85 years of age, with or without a diagnosis of COVID-19 confirmed according to the World Health Organization definition (laboratory-confirmed SARS-CoV-2 infection) (11) with symptoms severe enough to deserve hospital admission, and consecutively recruited from May to September 2020. Patients under mechanical ventilation at enrollment or who were unable to report the required data or to sign informed consent, or who were diagnosed with concurrent cancer, were excluded from the study. Control group was composed of hospitalized patients for reasons other than COVID-19, disease/disorders of gastroenterological, traumatic, and surgical pertinence, prospectively enrolled within study timeframe for the majority in Internal Medicine and in a minority in Gastroenterology Units within participating centers (see Supplementary Tables, Supplementary Digital Content 1, http://links.lww.com/AJG/C272).

### Study design

The study was performed in 36 centers in 12 countries: Italy, Bangladesh, Egypt, Israel, India, Macedonia, Romania, the Russian Federation, Serbia, Spain, Sweden, and Turkey. The country and center selection was based on the availability of interested country principal investigators contacted either directly by the coordinating center or responding spontaneously to advertisements of the study posted on the United European Gastroenterology, European Society of Neurogastroenterology and Motility, and Rome Foundation websites. Hospitalized patients with or without COVID-19 were prospectively and consecutively enrolled at hospital admission and followed up for 30 days to define disease outcome. The enrollment time frame lasted from 1 to 3 months for each center. Each center was asked to enroll 25 patients with and 25 without COVID-19.

### Assessment

Study data were simultaneously collected from each center using an e-Case report form on the REDCap platform. All demographics, medical history, laboratory and imaging tests, and other clinical data, including the presence of GI symptoms according to the Gastrointestinal Symptoms Rating Scale (GSRS) questionnaire at admission and a 1-month follow-up, were reported with descriptive statistics. All data present in the inpatient medical record were used for the study, including demographics, clinical parameters, age, sex, weight, height, body mass index, physical activity, alcohol intake, country of residence, ethnicity, medical history, chronic medication intake, routine blood examination, results of nasal/oropharyngeal swab for the diagnosis of COVID-19 infection, results of imaging examinations, and therapies administered during hospitalization. At admission, patients were assessed for the presence of COVID-19–related symptoms, including current or previous (1 week before hospitalization) GI symptoms, using the GSRS, which consists of 15 items including common upper and lower GI symptoms graded on a 7-item Likert-like scale (12). To assess the presence of chronic GI symptoms leading to overestimation of results, the presence of GI symptom onset at least 6 months before the hospitalization was assessed with the GSRS. After enrollment, all patients were subsequently contacted by telephone interview at 1 month to reassess GSRS and hospitalization outcomes (mechanical ventilation or need of ICU after interview, death, or main discharge diagnosis).

### End points

The primary end point of this study was the assessment of the prevalence of GI symptoms in hospitalized patients with COVID-19 compared with a non-COVID control group. Secondary end points included the identification of risk factors for the development of COVID-19–related GI symptoms and the persistence of GI symptoms 1 month after the initial assessment. Exploratory end points included symptom prevalence, univariate and multivariate analyses of predictive factors associated with GI symptoms, and symptom follow-up of the entire study cohort (1,961 patients).

### Statistical analysis

Continuous variables were reported as means and SD, and categorical variables as numbers and percentages. The presence of chronic GI symptoms was defined as the presence of at least 1 GSRS item with any degree of severity, except borborygmi, flatus, and eructation, for which the presence of at least 1 other GSRS item in association was required. Primary and secondary aim analyses were conducted after excluding subjects with chronic GI symptoms. Patients without COVID-19 diagnosis were used as control group for the primary study outcome. Data recorded at admission were compared using the χ^2^ test, Fisher test, and Student t-test when appropriate. These data were then tested as predictors of GI symptoms in patients with COVID-19 at admission and at questionnaire 1 month after initial assessment using logistic regression uni- and multivariate analyses. The estimated odds ratios with their 95% confidence intervals were calculated. The results obtained from the multivariate analysis were translated into graphic form, using a nomogram for logistic regression. *P* values less than 0.05 (2-tailed) were considered statistically significant. Survival among the groups of patients with and without GI symptoms was calculated using the Kaplan-Meier method and compared through the log-rank test. All analyses were performed using STATA statistical software (Stata Corp., College Station, TX). This study is registered with ClinicalTrials.gov, NCT04691895.

## RESULTS

### Patients

**Figure 1.**
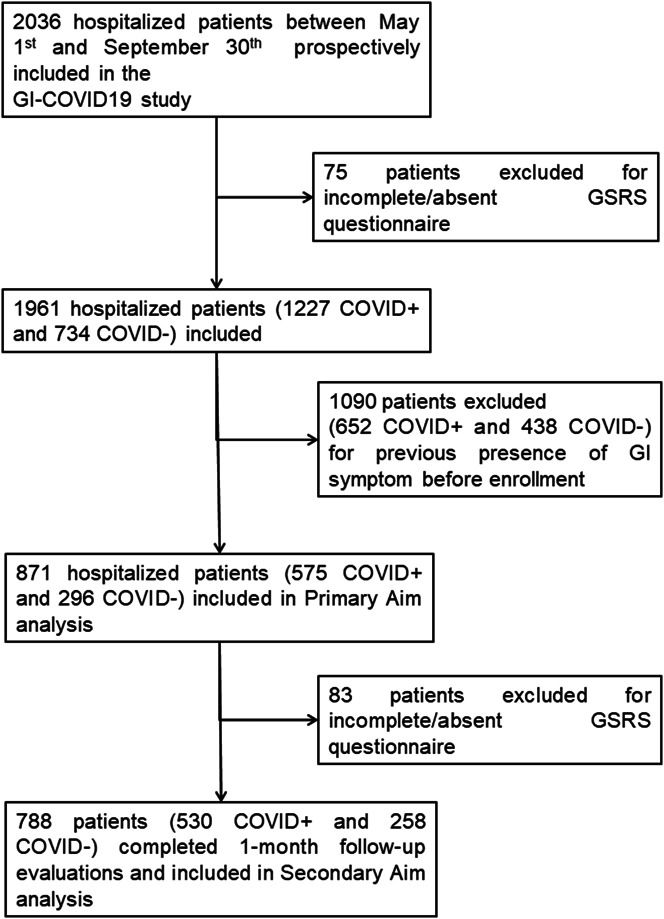
Flowchart of the selection of patients enrolled in the study. COVID-19, coronavirus-19 disease; GSRS, Gastrointestinal Symptoms Rating Scale.

From May 1 to September 30, 2020, a total of 2,036 hospitalized patients were consecutively enrolled in the study from the 36 recruiting centers. Of these, 75 patients were excluded for incomplete or absence of questionnaire data. Of the remaining 1,961 patients, 1,227 (62.6%) had a diagnosis of COVID-19. A total of 1,090 patients had pre-existing GI symptoms (652 in the COVID population and 438 in the control group) and were excluded from the primary and secondary aim analyses (Figure 1). Data of subjects without pre-existing GI symptoms (871 patients: 575 COVID-19 and 296 controls) were subsequently used to evaluate primary and secondary study aims. Demographic and clinical characteristics of patients included in the study are reported in Table 1. The control group diagnoses are detailed in the Supplementary Text (see Supplementary Digital Content 1, http://links.lww.com/AJG/C272). Overall, 17 patients died at the 1-month follow-up, of whom 11 had COVID-19 (3 without and 8 with GI symptoms) and 6 were controls (*P* = 0.868). For the exploratory end points, demographics, clinical history data, symptom prevalence, univariate and multivariate analyses of predictive factors associated with GI symptoms, and symptom follow-up of the entire study cohort (1,961 patients) are reported in Supplementary Tables 1–4 (see Supplementary Digital Content 2, 3, 4, and 5, respectively, http://links.lww.com/AJG/C272).

**Table 1.**
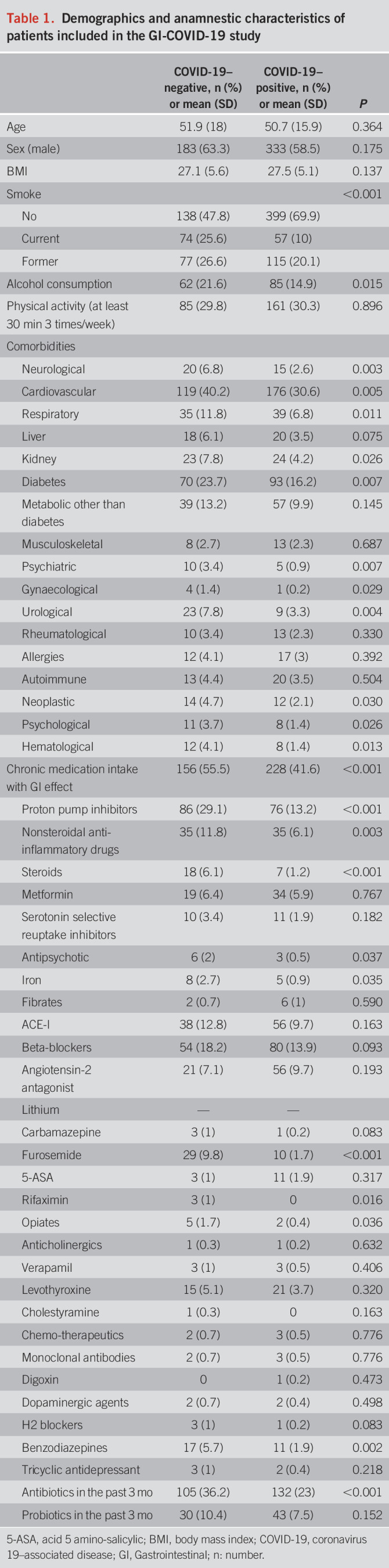
Demographics and anamnestic characteristics of patients included in the GI-COVID-19 study

### GI symptoms during COVID-19 infection

**Figure 2.**
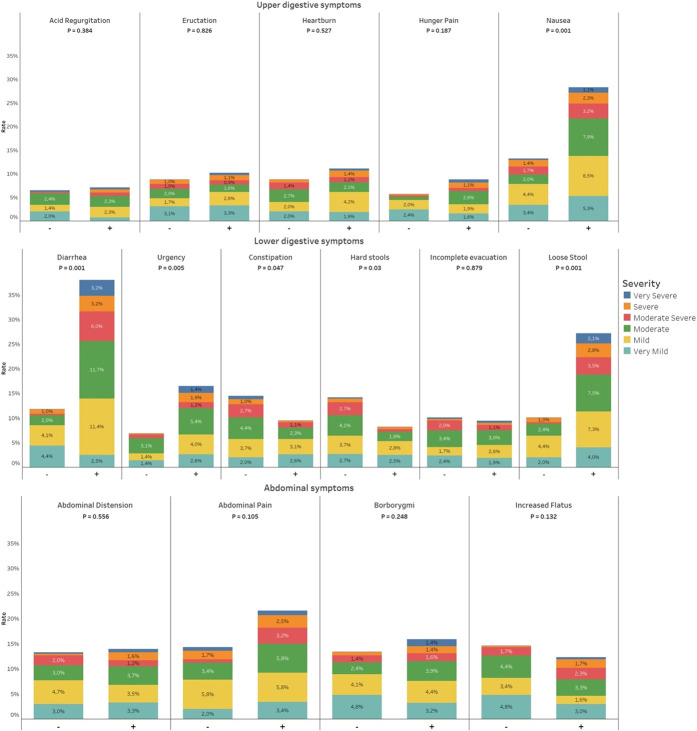
Gastrointestinal symptoms within the Gastrointestinal Symptom Rating Scale questionnaire complained by patients without (−) and with (+) COVID-19 diagnosis.

The overall prevalence of at least 1 GI symptom in the study population was 54.1% (471/871 patients). GI symptoms occurred more frequently in patients with COVID-19 (59.7%; 343/575 patients) than in the control group (43.2%; 128/296 patients) (*P* < 0.001). Patients with COVID-19, as compared with the control group, reported higher rates of nausea (28.2% vs 13.2%, *P* < 0.001), diarrhea (37.9% vs 11.9%, *P* < 0.001), loose stool (27.3% vs 10.2%, *P* < 0.001), and urgency (16.6% vs 6.8%, *P* = 0.005) and lower rates of constipation (9.4% vs 14.6%, *P* = 0.047) and hard stools (8.2% vs 14.2, *P* = 0.030) (Figure 2). Rates of GI symptom intensity scores in the study population are reported in Figure 2. The country distribution of the most prevalent GI symptom, namely, diarrhea, is reported in Supplementary Figure 1, Panel A (see Supplementary Digital Content 6, http://links.lww.com/AJG/C271).

### Risk factors associated with GI symptoms in patients with COVID-19

Clinical and laboratory data of the study population are detailed in Table 2. COVID-19 patients with GI symptoms, as compared with those without, had higher prevalence of symptoms such as fever (76.4% vs 58.6%, *P* < 0.001), fatigue (61.2% vs 50.4%, *P* = 0.010), myalgia (41.4% vs 29.7%, *P* = 0.005), dyspnea (35.6% vs 22.4%, *P* = 0.001), runny nose (10.5% vs 3%, *P* = 0.001), headache (35.3% vs 13.8%, *P* < 0.001), anosmia (30.3% vs 16.4%, *P* < 0.001), and dysgeusia (27.1% vs 14.7%, *P* < 0.001). When all COVID-positive patients were evaluated, patients with GI symptoms had higher levels of ferritin (mean 438 vs 392 ng/mL, *P* = 0.020) and lower levels of creatinine (mean 1.1 vs 1.2 mg/dL, *P* = 0.006). The needs for high-resolution chest computed tomography, mechanical ventilation, and ICU support were not statistically different between patients with and without GI symptoms. Similarly, Kaplan-Meier curves for 30-day survival showed no survival differences between patients with and without GI symptoms (log-rank test *P* = 0.338; see Supplementary Figure 2; see Supplementary Digital Content 7, http://links.lww.com/AJG/C271).

**Table 2.**
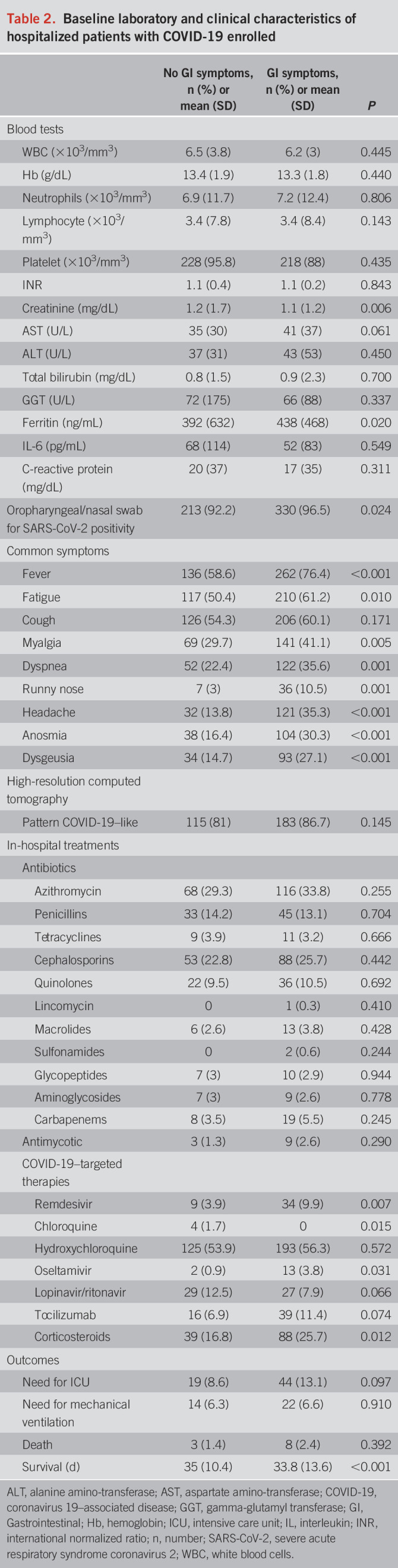
Baseline laboratory and clinical characteristics of hospitalized patients with COVID-19 enrolled

**Figure 3.**
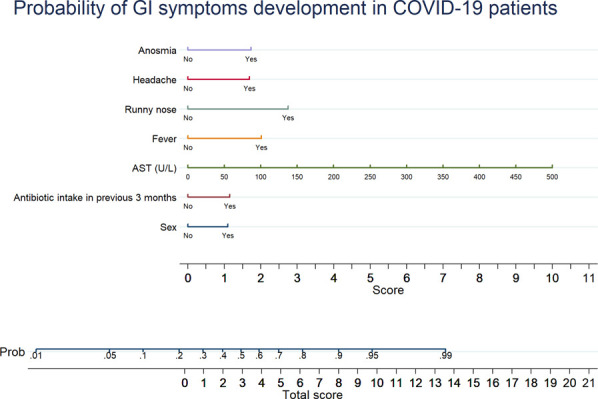
Nomogram reporting a probability score for GI symptoms development in patients with COVID-19. COVID-19, coronavirus-19 disease; GI, gastrointestinal.

Among factors associated with GI symptoms identified in univariate analysis, only 7 variables survived the multivariate analysis (see Supplementary Table 5, Supplementary Digital Content 8, http://links.lww.com/AJG/C272), namely, female sex, fever, headache, anosmia, runny nose, aspartate aminotransferase levels, and antibiotic intake within the 3 months before hospital admission. A nomogram assessing the individual risk of factors associated with GI symptoms is presented in Figure 3.

### Post-COVID-19 follow-up

#### GI symptoms at follow-up.

A total of 1,138 patients with COVID-19 and 645 controls completed the symptom questionnaire 1 month after initial assessment (response rates of 92.7% and 87.9%, respectively). Of these, 788 patients (530 with COVID-19 and 258 from the control group had no symptoms before hospitalization) were considered for further analyses (response rates of 92.2% and 87.2%, respectively). The prevalence of diarrhea at a 1-month follow-up in patients with COVID-19 for each country participating in the study is reported in Supplementary Figure 1, Panel B (see Supplementary Digital Content 6, http://links.lww.com/AJG/C271).

**Figure 4.**
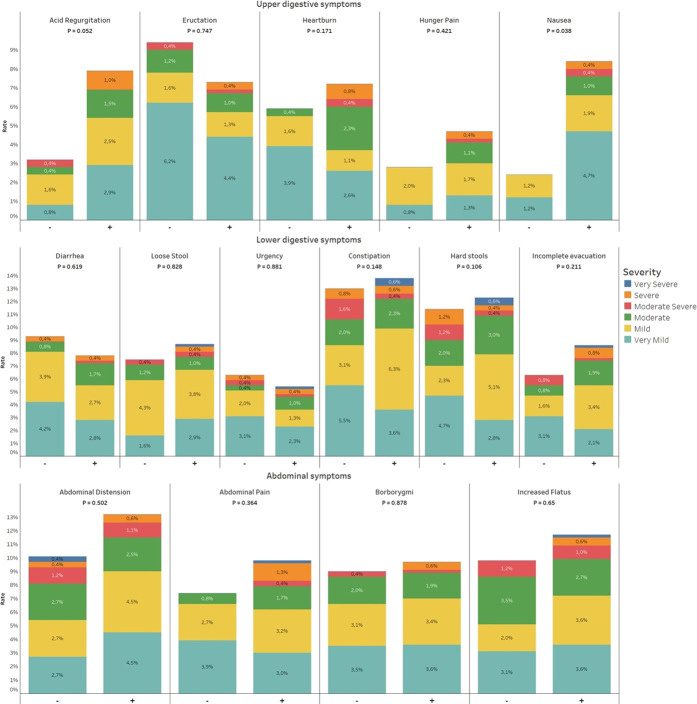
Variations in gastrointestinal symptoms within the Gastrointestinal Symptom Rating Scale questionnaire complained by patients without (−) and with (+) COVID-19 diagnosis after the 1-month follow-up.

At follow-up evaluation, nausea was significantly more prevalent in patients with COVID-19 than in the control group (8.3% vs 2.3%, *P* = 0.038). A borderline significantly higher prevalence was found for acid regurgitation (7.8% vs 3.1%, *P* = 0.052) (Figure 4). No other significant differences were found.

**Figure 5.**
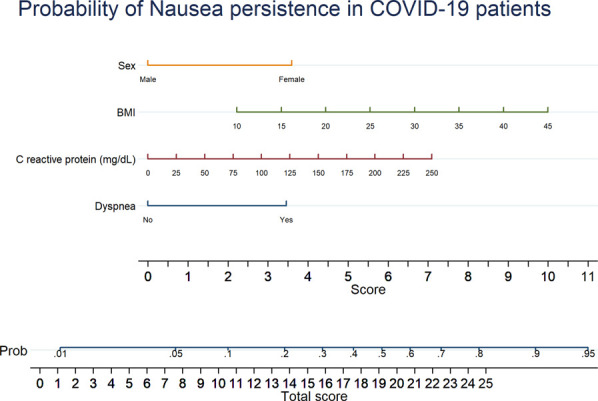
Nomogram reporting a probability score for nausea at 1 month after initial assessment in patients with COVID-19. BMI, body mass index; COVID-19, coronavirus-19 disease.

Factors associated with the presence of GI symptoms at 1 month after initial assessment identified in multivariate analysis are reported in Supplementary Table 6 (see Supplementary Digital Content 9, http://links.lww.com/AJG/C272). Among factors associated with nausea at 1 month after initial assessment identified in univariate analysis, only 4 variables survived the multivariate analysis (see Supplementary Table 7, Supplementary Digital Content 10, http://links.lww.com/AJG/C272), namely, female sex, body mass index, dyspnea, and C-reactive protein values. A nomogram assessing the individual risk of factors associated with nausea at 1 month is presented in Figure 5.

#### COVID-19 patients with symptoms at baseline.

In COVID-19 patients with at least 1 GI symptom at baseline, we found a significant reduction in the presence or intensity rates of most symptoms, except for eructation, constipation, hard stools, incomplete evacuation, abdominal distension, and flatus (see Supplementary Figure 3, Supplementary Digital Content 11, http://links.lww.com/AJG/C271). In particular, significant variations were found for hunger pain (14.4% at baseline vs 6% at follow-up), nausea (46.3% vs 10.2%), heartburn (18.1% vs 9.5%), acid regurgitation (12.2% vs 9.9%), diarrhea (63.9% vs 12.1%), loose stools (46% vs 12.8%), urgency (28.5% vs 7.6%), abdominal pain (35.6% vs 12.7%), and borborygmi (25.9% vs 13.4%).

#### COVID-19 patients without symptoms at baseline.

On the other hand, patients with COVID-19 without GI symptoms during the acute phase of SARS-CoV-2 infection developed GI symptoms at a 1-month follow-up in up to 9.8% of cases (see Supplementary Figure 4, Supplementary Digital Content 12, http://links.lww.com/AJG/C271). In particular, at a 1-month follow-up, we found a significant increase in the onset of eructation (4.7%), nausea (6%), acid regurgitation (4.7%), constipation (9.8%), hard stools (6.5%), abdominal pain (5.6%), and distension (9.3%).

## DISCUSSION

The GI-COVID-19 study provides evidence that SARS-CoV-2 infection is associated with diarrhea, nausea, and other GI symptoms, including loose stools and urgency, compared with a noninfected control population. Female patients with fever, runny nose, anosmia, headache, increased aspartate aminotransferase levels, and reporting antibiotic intake in the 3 months before hospital admission were at increased risk of GI symptoms. COVID-19 patients with GI symptoms had higher rates of systemic involvement, including fever, fatigue, myalgia, dyspnea, runny nose, headache, anosmia, and dysgeusia. However, the presence of GI symptoms did not increase the rate of ICU admission nor mortality. Although most GI symptoms declined at 1 month after the initial evaluation, the prevalence of nausea and acid regurgitation remained higher in the COVID-19 cohort of patients than in noninfected patients. Females with dyspnea, high body mass index, and increased C reactive protein values were more likely to report nausea at a 1-month follow-up.

The novelty of the GI-COVID-19 study stems from its inclusion of a control population of noninfected patients, its multicenter design, and the analysis of new gastrointestinal symptoms developed after acute SARS-CoV-2 infection, all features that have often been previously neglected. We also investigated clinical features, such as stool consistency and urgency, that have a high impact on patients' quality of life and have only been reported in a small retrospective uncontrolled study (13). All the features mentioned above may at least in part explain the higher prevalence of GI symptoms and diarrhea found in this study compared with pooled data reported in the literature (1,5,14), even when data were obtained from hospitalized patients or were adjusted for previous GI chronic diseases (15). Furthermore, our study included mostly western countries, in which comparative studies have found a higher prevalence of GI symptoms (15).

In addition, most of the abovementioned large studies were lacking a control group. Only small single-center studies (16,17) have reported the prevalence of GI symptoms in COVID-19 compared with a control group of nonhospitalized patients, but symptoms were recorded without the use of validated questionnaires.

Limited data are available regarding factors associated with GI symptoms. Our results are in line with previous multivariate analyses (18,19), finding that female sex and systemic or respiratory illness increased the risk for GI symptoms (18). In addition, recent reports (20,21) found that age and fever were the only significant predictive factors for GI symptoms at hospital admission.

Interestingly, our study showed that antibiotic treatment in the 3 months before hospitalization was associated with GI symptom development. These data suggest that antibiotic-associated dysbiosis and increased intestinal permeability may contribute to GI symptom development in the context of COVID-19 infection (22,23). The contribution of dysbiosis to the pathogenesis of COVID-19 is also supported by previous evidence showing enrichment of opportunistic pathogens along with depletion of commensals (22). The increased relative abundance of *Streptococcus*, *Rothia*, *Veillonella*, *Erysipelatoclostridium*, and *Actinomyces* may play a pathogenic role in COVID-19 infection because it has been associated with the magnitude of systemic inflammation (22). Proton pump inhibitors (PPIs) may also contribute to dysbiosis (22,23). A previous meta-analysis (24) found that patients taking PPIs had an increased risk of SARS-CoV-2 infection and worse outcomes. In this study, we also found a significant association of PPIs with GI symptom occurrence in multivariate analysis of the entire study cohort. However, in the primary aim population, this association was not confirmed.

We confirmed the results of previous large series (25,26) and pooled data (27,28) reporting no differences in ICU admission or mortality between patients with and without GI symptoms. The overall 1-month mortality of 1.9% in patients with COVID-19, irrespective of GI symptoms, found in our study is slightly higher than that previously reported. This is likely due to the inclusion of hospitalized patients and an older population compared with previous studies.

Few longitudinal studies have assessed changes in digestive symptoms over time in patients with COVID-19 (26,29). Carvalho-Schneider et al. (29) found no differences in the rate of diarrhea from enrollment to the 2-month follow-up in a small group of patients. By contrast, Luo et al. (26) showed a rapid disappearance of diarrhea and other related symptoms at a 3-week follow-up and a slow and sporadic appearance of symptoms in patients without digestive symptoms at enrollment. In this study, persistence of symptoms at a 1-month follow-up was found in less than 17% of cases, whereas among COVID-19–positive patients without GI symptoms at admission, 9.8% developed GI symptoms at follow-up. The only symptoms that were persistently more frequent in patients with COVID-19 than controls at follow-up were nausea and acid regurgitation. The persistence of these symptoms may be related to the neuroinvasive potential of COVID-19, which may lead to the presence of the virus in the dorsal vagal complex and the area postrema (4).

We acknowledge that our study has some limitations. First, the primary and secondary outcomes might have been influenced by a number of different factors in the study population, including the effects of therapies, hospitalization, and comorbidities. However, our effort to compare the COVID-positive patients with a control population of hospitalized patients likely mitigated the influence of these factors on the end points. Besides, it should be underlined that our COVID-19 cohort included only hospitalized patients consequently having a moderate to severe disease course, who, in turn, have been reported to complain high rates of GI symptoms (18), thus leading to discrepancies in GI symptoms occurrence rates when compared with the outpatient setting.

Second, the study network, comprising several gastroenterology wards, might have introduced a selection bias and influenced the outcome; nonetheless, excluding patients with chronic GI symptoms from the primary and secondary analyses might have sufficed to overcome this limitation.

In conclusion, the finding that COVID-19 is associated with GI symptoms in a high proportion of infected patients and the persistence of some of these symptoms may increase awareness of the role of GI involvement in COVID-19. Future studies should investigate whether treatment of GI symptoms during the acute phase can prevent long-lasting GI symptoms in COVID-19.

## CONFLICT OF INTEREST

**Guarantor of the article**: Giovanni Barbara, MD.

**Specific author contributions**: G.B., G.M., C.C., and V.S.: designed the study; G.M.: conducted statistical analysis; G.M., G.B., M.R.B., and C.C.: validated and interpretated data; G.B., G.M., M.R.B., C.C., and V.S.: drafted the article; all authors collected data for the study, critically revised and approved the final version of the article.

**Financial support**: G.B. contribution to this research was permitted in part by funding from Fondazione Cassa di Risparmio in Bologna; the Italian Ministry of Education, University and Research; and Fondazione del Monte di Bologna e Ravenna and European Grant H2020, DISCOvERIE, SC1-BHC-01-2019. M.R.B. is a recipient of a grant from the Italian Ministry of Health (Ricerca Finalizzata GR-2018-12367062). None of the funding organizations have had any role in the design and conduct of the study; in the collection, management, and analysis of the data; or in the preparation, review, and approval of the article.

**Potential competing interests**: None to report.

**Clinical trial number:** ClinicalTrials.gov number, NCT04691895.

**Study Highlights**

**WHAT IS KNOWN**

✓ SARS-CoV-2 may infect the gastrointestinal tract leading to nausea, vomiting, abdominal pain, and diarrhea.
✓ The attempts to evaluate the prevalence of gastrointestinal symptoms in SARS-CoV-2 infection gave high heterogeneous results.

**WHAT IS NEW HERE**

✓ Gastrointestinal symptoms occurred more frequently in patients with coronavirus-19 disease (COVID-19) (59.7%) than in the control group (43.2%).
✓ Patients with COVID-19 complained of higher presence or intensity of nausea, diarrhea, loose stools, and urgency as compared with controls.
✓ At a 1-month follow-up, nausea remained significantly increased in patients with COVID-19 over controls.
✓ Females with dyspnea, high body mass index, and C-reactive protein levels were more likely to report nausea at a 1-month follow-up.

## Supplementary Material
