# Supplementary material for: Prevalence of Gastrointestinal Symptoms in Severe Acute Respiratory Syndrome Coronavirus 2 Infection: Results of the Prospective Controlled Multinational GI-COVID-19 Study
Source: Am J Gastroenterol. 2021 Nov 9;117(1):147–57. doi: 10.14309/ajg.0000000000001541 (PMC10337314; doi:10.14309/ajg.0000000000001541)

**SUPPLEMENTAL FIGURES**

**Supplemental Figure 1 (Panel A and B).** Rate of diarrhea in COVID-19 patients for each country participating in the study A) at baseline and B) at 1-month follow-up (rates of Sweden and India are not displayed due to the small sample size).


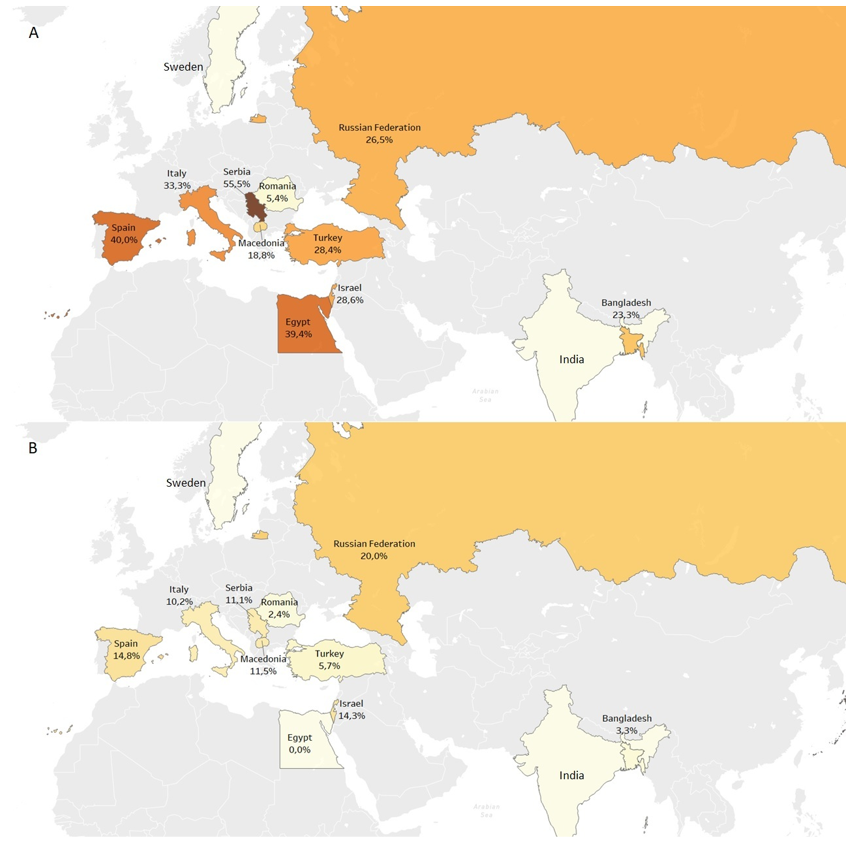


**Supplemental Figure 2.** Kaplan-Meier curves for overall survival of COVID-19 patients according to gastrointestinal symptoms presence.


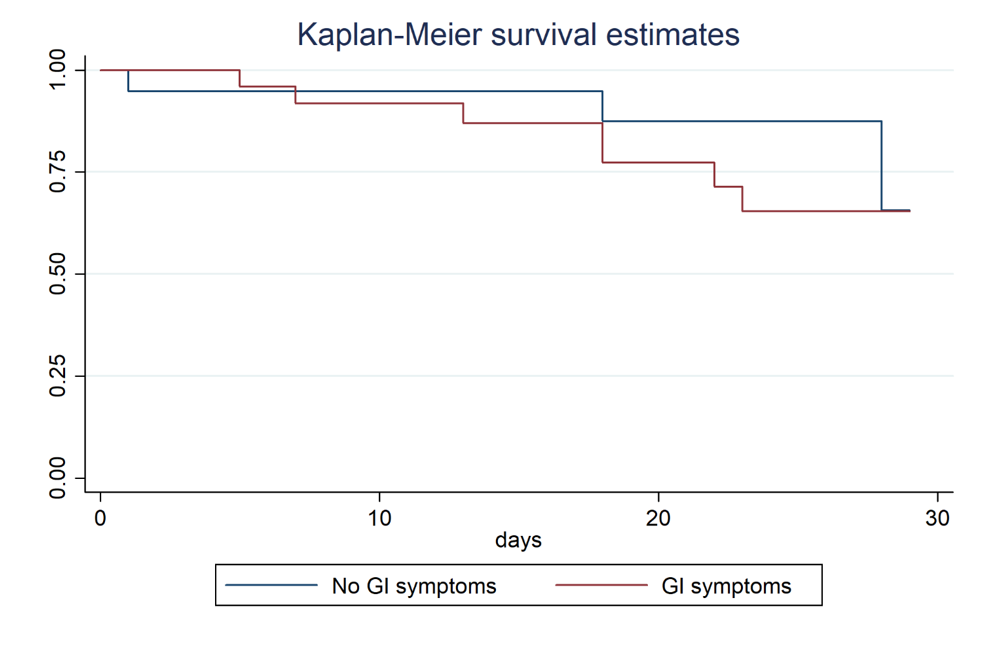


**Supplemental Figure 3.** Variations in gastrointestinal symptoms within the GSRS questionnaire complained by COVID-19 patients referring gastrointestinal symptoms at hospital admission and after 1-month follow-up.


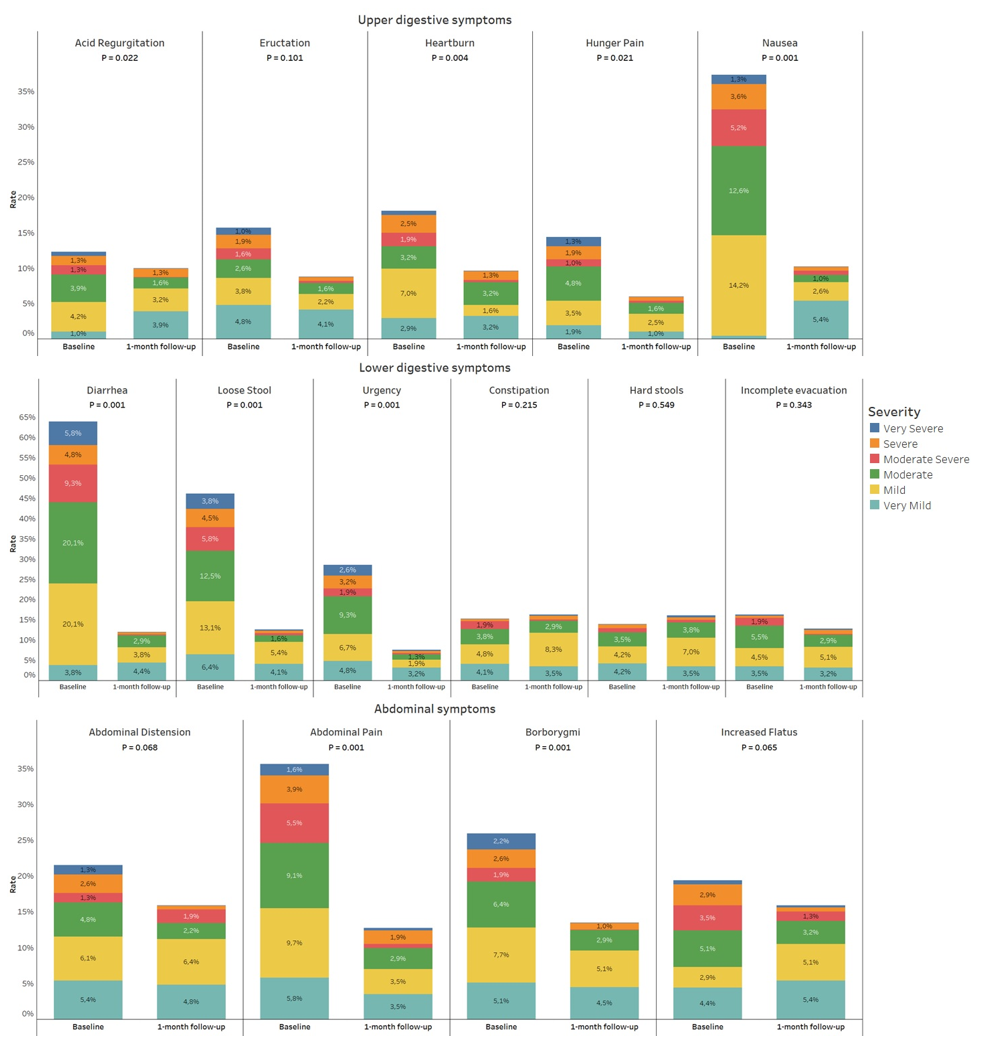


**Supplemental Figure 4.** Variations in gastrointestinal symptoms within the GSRS questionnaire complained by COVID-19 patients not referring gastrointestinal symptoms at hospital admission and after 1-month follow-up.


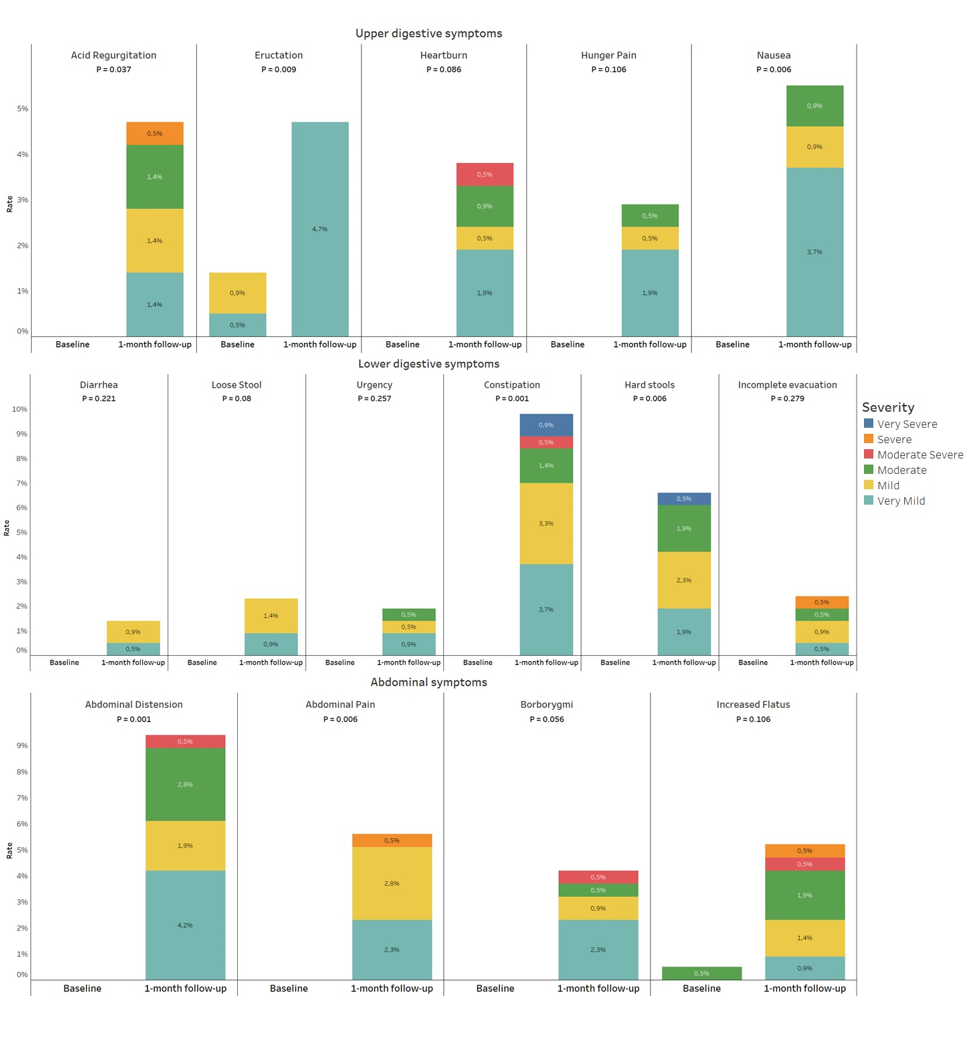

Supplement: Supplementary file 1 [file acg-117-147-s001.docx]
