# Supplementary material for: Prevalence of Gastrointestinal Symptoms in Severe Acute Respiratory Syndrome Coronavirus 2 Infection: Results of the Prospective Controlled Multinational GI-COVID-19 Study
Source: Am J Gastroenterol. 2021 Nov 9;117(1):147–57. doi: 10.14309/ajg.0000000000001541 (PMC10337314; doi:10.14309/ajg.0000000000001541)
Supplement: Supplementary file 2 [file acg-117-147-s002.docx]

**Supplemental Text**

Control group (COVID-19 negative patients) was composed by 296 hospitalized patients prospectively enrolled in Internal Medicine units within participating centers, admitted and thereafter discharged for the following diseases/disorders other than trauma and surgery, by specialty pertinence: neurological (n. 20), cardiovascular (n. 63), respiratory (n. 36), liver (n. 13), kidney (n. 14), diabetes (n. 14), metabolic (n. 3), psychiatric and psychological (n. 3), gynaecological (n. 9), urological (n. 12), rheumatological (n. 6), autoimmune (n. 5), haematological (n. 4), other (n. 58).

**Supplemental Table 1.** Demographics and anamnestic characteristics of the global study population included in the study.

|  | **COVID-19 negative**  n (%) or Mean (SD) | **COVID-19 positive**  n (%) or Mean (SD) | **p** |
| --- | --- | --- | --- |
| **Age** | 54.2 (17.8) | 50.6 (16) | <0.001 |
| **Sex (Male)** | 401 (55.7) | 655 (53.8) | 0.424 |
| **BMI** | 27 (5.9) | 27.5 (5.4) | 0.012 |
| **Smoke** |  |  | <0.001 |
| No | 357 (49.5) | 849 (69.8) |  |
| Current | 171 (23.7) | 132 (10.9) |  |
| Former | 194 (26.9) | 235 (19.3) |  |
| **Alcohol consumption** | 146 (20.3) | 164 (13.5) | <0.001 |
| **Physical activity (at least 30 minutes 3 times/week)** | 226 (31.6) | 351 (30.3) | 0.555 |
| **Comorbidities** |  |  |  |
| Neurological | 81 (11) | 44 (3.6) | <0.001 |
| Cardiovascular | 318 (43.3) | 368 (30) | <0.001 |
| Respiratory | 100 (13.6) | 107 (8.7) | 0.001 |
| Liver | 49 (6.7) | 36 (2.9) | <0.001 |
| Kidney | 88 (12) | 67 (5.5) | <0.001 |
| Diabetes | 175 (23.8) | 206 (16.8) | <0.001 |
| Metabolic other than diabetes | 119 (16.2) | 136 (11.1) | <0.001 |
| Musculoskeletal | 43 (5.9) | 35 (2.9) | 0.001 |
| Psychiatric | 25 (3.4) | 20 (1.6) | 0.011 |
| Gynaecological | 13 (1.8) | 7 (0.6) | 0.010 |
| Urological | 47 (6.4) | 40 (3.3) | 0.001 |
| Rheumatological | 35 (4.8) | 38 (3.1) | 0.058 |
| Allergies | 34 (4.6) | 41 (3.3) | 0.149 |
| Autoimmune | 32 (4.4) | 117 (9.5) | <0.001 |
| Neoplastic | 26 (3.5) | 31 (2.5) | 0.195 |
| Psychological | 40 (5.5) | 19 (1.6) | <0.001 |
| Haematological | 33 (4.5) | 33 (2.7) | 0.032 |
| **Chronic medication intake with GI effect** | 430 (64.1) | 571 (48.7) | <0.001 |
| Proton pump inhibitors | 271 (36.9) | 224 (18.3) | <0.001 |
| Non-steroidal anti-inflammatory drugs | 99 (13.5) | 78 (6.4) | <0.001 |
| Steroids | 43 (5.9) | 32 (2.6) | <0.001 |
| Metformin | 55 (7.5) | 81 (6.6) | 0.452 |
| Serotonin selective re-uptake inhibitors | 43 (5.9) | 32 (2.6) | <0.001 |
| Antipsychotic | 15 (2) | 10 (0.8) | 0.019 |
| Iron | 20 (2.7) | 10 (0.8) | 0.001 |
| Fibrates | 8 (1.1) | 11 (0.9) | 0.672 |
| ACE-I | 100 (13.6) | 114 (9.3) | 0.003 |
| Beta-blockers | 145 (19.8) | 153 (12.5) | <0.001 |
| Angiotensin-2 antagonist | 89 (12.1) | 109 (8.9) | 0.021 |
| Lithium | 1 (0.1) | 1 (0.1) | 0.713 |
| Carbamazepine | 4 (0.5) | 3 (0.2) | 0.280 |
| Furosemide | 77 (10.5) | 19 (1.6) | <0.001 |
| 5-ASA | 14 (1.9) | 87 (7.1) | <0.001 |
| Rifaximin | 8 (1.1) | 2 (0.2) | 0.005 |
| Opiates | 18 (2.5) | 7 (0.6) | <0.001 |
| Anticholinergics | 8 (1.1) | 5 (0.4) | 0.072 |
| Verapamil | 4 (0.5) | 3 (0.2) | 0.280 |
| Levothyroxine | 54 (7.4) | 66 (5.4) | 0.077 |
| Cholestyramine | 2 (0.3) | 1 (0.1) | 0.295 |
| Chemotherapeutics | 5 (0.7) | 15 (1.2) | 0.248 |
| Monoclonal antibodies | 5 (0.7) | 36 (2.9) | 0.001 |
| Digoxin | 3 (0.4) | 4 (0.3) | 0.766 |
| Dopaminergic agents | 4 (0.5) | 2 (0.2) | 0.138 |
| H2 blockers | 8 (1.1) | 5 (0.4) | 0.072 |
| Benzodiazepines | 30 (5.3) | 21 (1.7) | <0.001 |
| Tricyclic antidepressant | 22 (3) | 6 (0.5) | <0.001 |
| Antibiotics in the last 3 months | 254 (35) | 364 (29.7) | 0.016 |
| Probiotics in the last 3 months | 84 (11.6) | 121 (9.9) | 0.234 |

*Abbreviations: COVID-19: coronavirus 19 associated disease; n: number; SD: standard deviation; 5-ASA: acid 5 amino-salicylic.*

**Supplemental Table 2.** Presence of gastrointestinal symptoms according to GSRS during the week before hospital admission in the global study population included in the study.

|  | **No** | **Very Mild** | **Mild** | **Moderate** | **Moderate Severe** | **Severe** | **Very Severe** | **p** |
| --- | --- | --- | --- | --- | --- | --- | --- | --- |
| **Upper digestive symptoms** |  |  |  |  |  |  |  |  |
| Acid regurgitation |  |  |  |  |  |  |  | 0.041 |
| COVID-19 negative | 561 (76.5) | 45 (6.1) | 49 (6.7) | 47 (6.4) | 17 (2.3) | 11 (1.5) | 3 (0.4) |  |
| COVID-19 positive | 998 (81.8) | 46 (3.8) | 76 (6.2) | 61 (5) | 14 (1.2) | 18 (1.5) | 7 (0.6) |  |
| Eructation |  |  |  |  |  |  |  | 0.005 |
| COVID-19 negative | 538 (74) | 59 (8.1) | 50 (6.9) | 42 (5.8) | 17 (2.3) | 16 (2.2) | 5 (0.7) |  |
| COVID-19 positive | 990 (81.1) | 54 (4.4) | 73 (6) | 50 (4.1) | 29 (2.4) | 17 (1.4) | 8 (0.7) |  |
| Heartburn |  |  |  |  |  |  |  | 0.168 |
| COVID-19 negative | 552 (75.4) | 42 (5.7) | 55 (7.5) | 48 (6.6) | 19 (2.6) | 13 (1.8) | 3 (0.4) |  |
| COVID-19 positive | 983 (80.4) | 56 (4.6) | 69 (5.7) | 58 (4.8) | 25 (2.1) | 23 (1.9) | 8 (0.7) |  |
| Hunger pain |  |  |  |  |  |  |  | 0.053 |
| COVID-19 negative | 603 (82.6) | 41 (5.6) | 38 (5.2) | 32 (4.4) | 13 (1.8) | 2 (0.3) | 1 (0.1) |  |
| COVID-19 positive | 1006 (82.5) | 65 (5.3) | 49 (4) | 55 (4.5) | 16 (1.3) | 21 (1.7) | 7 (0.6) |  |
| Nausea |  |  |  |  |  |  |  | 0.133 |
| COVID-19 negative | 520 (71.3) | 42 (5.8) | 67 (9.2) | 58 (8) | 17 (2.3) | 19 (2.6) | 6 (0.8) |  |
| COVID-19 positive | 798 (65.6) | 81 (6.7) | 119 (9.8) | 108 (8.9) | 47 (3.9) | 46 (3.8) | 17 (1.4) |  |
| **Lower digestive symptoms** |  |  |  |  |  |  |  |  |
| Diarrhea |  |  |  |  |  |  |  | <0.001 |
| COVID-19 negative | 562 (77) | 43 (5.9) | 59 (8.1) | 39 (5.3) | 10 (1.4) | 16 (2.2) | 1 (0.1) |  |
| COVID-19 positive | 662 (54.2) | 87 (7.1) | 150 (12.3) | 178 (14.6) | 62 (5.1) | 48 (3.9) | 35 (2.9) |  |
| Loose stool |  |  |  |  |  |  |  | <0.001 |
| COVID-19 negative | 581 (79.4) | 38 (5.2) | 50 (6.8) | 40 (5.5) | 7 (1) | 16 (2.2) | 0 |  |
| COVID-19 positive | 797 (65.3) | 80 (6.6) | 111 (9.1) | 117 (9.6) | 50 (4.1) | 41 (3.4) | 25 (2.1) |  |
| Urgency |  |  |  |  |  |  |  | 0.001 |
| COVID-19 negative | 633 (86.7) | 27 (3.7) | 24 (3.3) | 26 (3.6) | 13 (1.8) | 7 (1) | 0 |  |
| COVID-19 positive | 985 (80.7) | 42 (3.4) | 57 (4.7) | 84 (6.9) | 20 (1.6) | 17 (1.4) | 15 (1.2) |  |
| Constipation |  |  |  |  |  |  |  | <0.001 |
| COVID-19 negative | 486 (66.5) | 32 (4.4) | 68 (9.3) | 68 (9.3) | 42 (5.8) | 22 (3) | 13 (1.8) |  |
| COVID-19 positive | 967 (79) | 52 (4.3) | 64 (5.2) | 79 (6.5) | 34 (2.8) | 26 (2.1) | 2 (0.2) |  |
| Hard stools |  |  |  |  |  |  |  | <0.001 |
| COVID-19 negative | 506 (69.3) | 31 (4.3) | 61 (8.4) | 64 (8.8) | 36 (4.9) | 18 (2.5) | 14 (1.9) |  |
| COVID-19 positive | 991 (81.3) | 50 (4.1) | 53 (4.4) | 73 (6) | 27 (2.2) | 22 (1.8) | 3 (0.3) |  |
| Incomplete evacuation |  |  |  |  |  |  |  | 0.100 |
| COVID-19 negative | 570 (78.2) | 34 (4.7) | 41 (5.6) | 43 (5.9) | 25 (3.4) | 11 (1.5) | 5 (0.7) |  |
| COVID-19 positive | 1011 (83.1) | 42 (3.5) | 65 (5.3) | 52 (4.3) | 22 (1.8) | 18 (1.5) | 7 (0.6) |  |
| **Abdominal symptoms** |  |  |  |  |  |  |  |  |
| Abdominal distension |  |  |  |  |  |  |  | 0.025 |
| COVID-19 negative | 472 (64.6) | 44 (6) | 74 (10.1) | 69 (9.4) | 36 (4.9) | 21 (2.9) | 15 (2.1) |  |
| COVID-19 positive | 841 (68.8) | 95 (7.8) | 101 (8.3) | 110 (9) | 33 (2.7) | 29 (2.4) | 14 (1.1) |  |
| Abdominal pain |  |  |  |  |  |  |  | 0.001 |
| COVID-19 negative | 488 (66.6) | 36 (4.9) | 82 (11.2) | 77 (10.5) | 21 (2.9) | 19 (2.6) | 10 (1.4) |  |
| COVID-19 positive | 806 (66.5) | 120 (9.9) | 107 (8.8) | 94 (7.8) | 44 (3.6) | 32 (2.6) | 9 (0.7) |  |
| Borborygmi |  |  |  |  |  |  |  | 0.042 |
| COVID-19 negative | 526 (72.1) | 57 (7.8) | 69 (9.5) | 36 (4.9) | 23 (3.2) | 17 (2.3) | 2 (0.3) |  |
| COVID-19 positive | 872 (71.4) | 97 (7.9) | 99 (8.1) | 89 (7.3) | 32 (2.6) | 17 (1.4) | 16 (1.3) |  |
| Increased flatus |  |  |  |  |  |  |  | <0.001 |
| COVID-19 negative | 501 (68.5) | 45 (6.2) | 59 (8.1) | 81 (11.1) | 25 (3.4) | 17 (2.3) | 3 (0.4) |  |
| COVID-19 positive | 947 (77.4) | 61 (5) | 60 (4.9) | 82 (6.7) | 34 (2.8) | 30 (2.5) | 10 (0.8) |  |

**Supplemental Table 3.** Baseline laboratory and clinical characteristics of hospitalized COVID-19 patients enrolled according to the global study population.

|  | **No GI symptoms**  n (%) or Mean (SD) | **GI symptoms**  n (%) or Mean (SD) | **p** |
| --- | --- | --- | --- |
| **Blood tests** |  |  |  |
| WBC (*10^3^/mm^3^) | 6.44 (3.68) | 6.37 (2.95) | 0.768 |
| Hb (g/dL) | 13.4 (1.86) | 13.2 (1.84) | 0.069 |
| Neutrophils (*10^3^/mm^3^) | 6.61 (11.26) | 6.46 (10.99) | 0.856 |
| Lymphocyte (*10^3^/mm^3^) | 3.23 (7.48) | 2.90 (6.89) | 0.519 |
| Platelet (*10^3^/mm^3^) | 229 (96) | 233 (133) | 0.615 |
| INR | 1.09 (0.38) | 1.06 (0.17) | 0.212 |
| Creatinine (mg/dL) | 1.21 (1.66) | 1.08 (1.27) | 0.160 |
| AST (U/L) | 36 (30) | 36 (29) | 0.812 |
| ALT (U/L) | 39 (32) | 37 (40) | 0.603 |
| Total bilirubin (mg/dL) | 0.80 (1.44) | 0.79 (1.65) | 0.910 |
| GGT (U/L) | 72 (171) | 55 (77) | 0.077 |
| Ferritin (ng/mL) | 401 (635) | 354 (479) | 0.283 |
| IL-6 (pg(mL) | 68 (113) | 47 (72) | 0.125 |
| C reactive protein (mg/dL) | 23 (40) | 17 (38) | 0.052 |
| **Oropharyngeal/Nasal swab for SARS-CoV-2 positivity** | 235 (92.9) | 871 (94.3) | 0.414 |
| **Common symptoms** |  |  |  |
| Fever | 145 (57.1) | 612 (66) | 0.009 |
| Fatigue | 126 (49.6) | 588 (63.4) | <0.001 |
| Cough | 135 (53.2) | 508 (54.8) | 0.640 |
| Myalgia | 74 (29.1) | 357 (38.5) | 0.006 |
| Dyspnoea | 59 (23.2) | 304 (32.8) | 0.003 |
| Runny nose | 9 (3.5) | 83 (9) | 0.004 |
| Headache | 31 (12.2) | 269 (29) | <0.001 |
| Anosmia | 45 (17.7) | 230 (24.8) | 0.018 |
| Dysgeusia | 40 (15.8) | 205 (22.1) | 0.027 |
| **HRCT** | 164 (68.3) | 689 (76.8) | 0.007 |
| HRCT with pattern COVID-19 like | 124 (79.5) | 547 (82.3) | 0.421 |
| **In-hospital treatments** |  |  |  |
| ***Antibiotics*** | 153 (60.7) | 590 (63.7) | 0.393 |
| Azytromicin | 70 (27.6) | 312 (33.7) | 0.066 |
| Penicillins | 39 (15.4) | 92 (9.9) | 0.015 |
| Tetracyclines | 9 (3.5) | 18 (1.9) | 0.130 |
| Cephalosporins | 57 (22.4) | 217 (23.4) | 0.746 |
| Quinolones | 21 (8.3) | 107 (11.5) | 0.137 |
| Lincomycin | 0 | 1 (0.1) | 0.601 |
| Macrolides | 6 (2.4) | 24 (2.6) | 0.839 |
| Sulfonamides | 0 | 3 (0.3) | 0.364 |
| Glycopeptides | 7 (2.8) | 22 (2.4) | 0.727 |
| Aminoglycosides | 7 (2.8) | 16 (1.7) | 0.293 |
| Carbapenems | 9 (3.5) | 35 (3.8) | 0.862 |
| ***Antimycotic*** | 4 (1.6) | 21 (2.3) | 0.528 |
| ***COVID-19 targeted therapies*** | 206 (81.1) | 738 (79.8) | 0.641 |
| Remdesivir | 10 (3.9) | 52 (5.6) | 0.290 |
| Chloroquine | 4 (1.6) | 9 (1) | 0.414 |
| Hydroxychloroquine | 140 (55.1) | 540 (58.3) | 0.371 |
| Oseltamivir | 3 (1.2) | 52 (5.6) | 0.003 |
| Lopinavir/Ritonavir | 43 (16.9) | 55 (5.9) | <0.001 |
| Tocilizumab | 17 (6.7) | 65 (7) | 0.859 |
| Corticosteroids | 40 (15.8) | 152 (16.4) | 0.804 |
| **Outcomes** |  |  |  |
| Need for ICU | 20 (8.3) | 85 (9.4) | 0.579 |
| Need for mechanical ventilation | 15 (6.2) | 34 (3.8) | 0.098 |
| Death | 3 (1.2) | 11 (1.2) | 0.980 |
| Survival (days) | 35.4 (10.7) | 34.9 (12.7) | 0.069 |

*Abbreviations: COVID-19: coronavirus 19 associated disease; n: number; SD: standard deviation; WBC: white blood cells; Hb: hemoglobin; INR: international normalized ratio; AST: aspartate amino-transferase; ALT: alanine amino-transferase; GGT: gamma-glutamyl transferase; IL: interleukin; SARS-CoV-2: severe acute respiratory syndrome coronavirus 2.*

# **Supplemental Table 4.** Uni- and multivariate logistic regression for identifying factors associated with gastrointestinal symptoms in COVID-19 patients according to the global study population.

|  | ***Univariate*** | | ***Multivariate*** | |
| --- | --- | --- | --- | --- |
|  | **OR (95% CI)** | ***p*** | **OR (95% CI)** | ***p*** |
| Sex (Male) | 0.607 (0.456-0.808) | 0.001 | 0.577 (0.427-0.779) | <0.001 |
| Physical activity | 1.990 (1.403-2.821) | <0.001 |  |  |
| **Comorbidities** |  |  |  |  |
| Autoimmune diseases | 3.440 (1.715-6.898) | 0.001 |  |  |
| Haematological diseases | 4.069 (0.0964-17.169) | 0.056 |  |  |
| **Chronic medication intake** |  |  |  |  |
| Proton pump inhibitors | 1.730 (1.155-2.590) | 0.008 | 1.739 (1.143-2.645) | 0.010 |
| 5-ASA | 2.361 (1.165-4.786) | 0.017 | 3.950 (1.899-8.217) | <0.001 |
| Antibiotic intake in the previous 3 months | 2.114 (1.497-2.986) | <0.001 | 1.867 (1.307-2.669) | 0.001 |
| Probiotic intake in the previous 3 months | 2.666 (1.443-4.923) | <0.001 |  |  |
| **Blood tests** |  |  |  |  |
| Haemoglobin | 0.930 (0.860-1.006) | 0.069 |  |  |
| GGT* | 0.999 (0.997-1.000) | 0.098 |  |  |
| C reactive protein | 0.997 (0.993-1.000) | 0.055 |  |  |
| **Common symptoms** |  |  |  |  |
| Fever | 1.460 (1.100-1.939) | 0.009 | 1.440 (1.061-1.956) | 0.019 |
| Fatigue | 1.762 (1.332-2.331) | <0.001 |  |  |
| Myalgia | 1.523 (1.127-2.059) | 0.006 |  |  |
| Dyspnoea | 1.614 (1.169-2.225) | 0.004 | 1.423 (1.021-2.009) | 0.038 |
| Runny nose | 2.677 (1.326-5.404) | 0.006 |  |  |
| Headache | 2.940 (1.968-4.394) | <0.001 | 2.939 (1.946-4.437) | <0.001 |
| Anosmia | 1.532 (1.075-2.185) | 0.018 |  |  |
| Dysgeusia | 1.519 (1.047-2.203) | 0.027 |  |  |

*per unit increase

*Abbreviations: COVID-19: coronavirus 19 associated disease; OR: odd ratio; CI: confidence interval; AST: aspartate amino-transferase; SARS-CoV-2: severe acute respiratory syndrome coronavirus 2.*

**Supplemental Table 5.** Uni- and multivariate logistic regression for identifying factors associated with gastrointestinal symptoms in COVID-19 patients.

|  | ***Univariate*** | | ***Multivariate*** | |
| --- | --- | --- | --- | --- |
|  | **OR (95% CI)** | ***p*** | **OR (95% CI)** | ***p*** |
| Sex (Female) | 1.387 (0.984-1.954) | 0.061 | 1.605 (1.075-2.398) | 0.021 |
| **Chronic medication intake** |  |  |  |  |
| Antibiotic intake in the previous 3 months | 1.622 (1.074-2.449) | 0.021 | 1.641 (1.014-2.655) | 0.044 |
| Probiotic intake in the previous 3 months | 2.379 (1.148-4.927) | 0.020 |  |  |
| **Blood tests** |  |  |  |  |
| AST (U/L) * | 1.006 (0.999-1.013) | 0.056 | 1.009 (1.002-1.016) | 0.017 |
| Oropharyngeal/Nasal swab positive for SARS-CoV-2 | 2.323 (1.097-4.922) | 0.028 |  |  |
| **Common symptoms** |  |  |  |  |
| Fever | 2.283 (1.591-3.276) | <0.001 | 2.389 (1.573-3.625) | <0.001 |
| Fatigue | 1.552 (1.108-2.173) | 0.011 |  |  |
| Myalgia | 1.649 (1.157-2.350) | 0.006 |  |  |
| Dyspnea | 1.911 (1.307-2.793) | 0.001 |  |  |
| Runny nose | 3.769 (1.647-8.624) | 0.002 | 3.280 (1.270-8.473) | 0.014 |
| Headache | 3.407 (2.207-5.257) | <0.001 | 2.077 (1.270-3.398) | 0.004 |
| Anosmia | 2.221 (1.464-3.371) | <0.001 | 2.118 (1.249-3.591) | 0.005 |
| Dysgeusia | 2.166 (1.402-3.346) | <0.001 |  |  |

*per unit increase

*Abbreviations: COVID-19: coronavirus 19 associated disease; OR: odd ratio; CI: confidence interval; AST: aspartate amino-transferase; SARS-CoV-2: severe acute respiratory syndrome coronavirus 2.*

**Supplemental Table 6.** Uni- and multivariate logistic regression for identifying factors associated with gastrointestinal symptoms persistence at follow-up in COVID-19 patients.

|  | ***Univariate*** | | ***Multivariate*** | |
| --- | --- | --- | --- | --- |
|  | **OR (95% CI)** | ***p*** | **OR (95% CI)** | ***p*** |
| Alcohol habits | 1.826 (1.123-2.967) | 0.015 | 1.883 (1.145-3.094) | 0.013 |
| **Chronic medication intake** |  |  |  |  |
| Antibiotic intake in the previous 3 months | 1.740 (1.158-2.614) | 0.008 | 1.646 (1.081-2.507) | 0.020 |
| Probiotic intake in the previous 3 months | 2.612 (1.359-5.023) | 0.004 |  |  |
| **Common symptoms** |  |  |  |  |
| Fever | 1.408 (0.959-2.066) | 0.080 |  |  |
| Fatigue | 1.357 (0.952-1.932) | 0.091 |  |  |
| Dyspnea | 1.574 (1.062-2.252) | 0.023 |  |  |
| Cough | 1.372 (0.961-1.958) | 0.082 |  |  |
| Headache | 1.866 (1.263-2.756) | 0.002 | 1.711 (1.147-2.552) | 0.008 |
| **Hospitalization-related factors** |  |  |  |  |
| Azitromycin | 1.368 (0.943-1.982) | 0.098 |  |  |
| Remdesivir | 2.299 (1.074-4.920) | 0.032 |  |  |
| Tocilizumab | 2.349 (1.286-4.292) | 0.005 | 2.185 (1.177-4.056) | 0.013 |
| Need for ICU | 1.696 (0.940-3.059) | 0.079 |  |  |

*per unit increase

*Abbreviations: COVID-19: coronavirus 19 associated disease; OR: odd ratio; CI: confidence interval; AST: aspartate amino-transferase; SARS-CoV-2: severe acute respiratory syndrome coronavirus 2; ICU: Intensive care unit.*

**Supplemental Table 7.** Uni- and multivariate logistic regression for identifying factors associated with nausea persistence at follow-up in COVID-19 patients.

|  | ***Univariate*** | | ***Multivariate*** | |
| --- | --- | --- | --- | --- |
|  | **OR (95% CI)** | ***p*** | **OR (95% CI)** | ***p*** |
| Sex (Female) | 2.411 (1.279-4.542) | 0.006 | 2.501 (1.300-4.813) | 0.006 |
| BMI* | 1.067 (1.011-1.125) | 0.018 | 1.058 (1.004-1.116) | 0.037 |
| **Chronic medication intake** |  |  |  |  |
| TCA | 11.279 (0.693-183.506) | 0.089 |  |  |
| Probiotic intake in the previous 3 months | 3.051 (1.312-7.092) | 0.010 |  |  |
| **Common symptoms** |  |  |  |  |
| Dyspnea | 2.496 (1.339-4.654) | 0.004 | 2.416 (1.273-4.587) | 0.007 |
| Runny nose | 3.051 (1.312-7.092) | 0.010 |  |  |
| **Blood tests** |  |  |  |  |
| Total bilirubin (mg/dL)* | 1.149 (0.979-1.348) | 0.088 |  |  |
| C reactive protein (mg/dL)* | 1.007 (0.999-1.014) | 0.053 | 1.007 (1.000-1.015) | 0.049 |
| **Hospitalization-related factors** |  |  |  |  |
| Cephalosporins | 0.362 (0.140-0.940) | 0.037 |  |  |

*per unit increase

*Abbreviations: COVID-19: coronavirus 19 associated disease; OR: odd ratio; CI: confidence interval; AST: aspartate amino-transferase; SARS-CoV-2: severe acute respiratory syndrome coronavirus 2; ICU: Intensive care unit.*
